# Effects of Selective Retrieval Practice on Older Adults: Lesser Benefits, Greater Losses

**DOI:** 10.3390/bs15030308

**Published:** 2025-03-05

**Authors:** Shaohang Liu, Christopher Kent, Josie Briscoe

**Affiliations:** 1 Institute of Developmental Psychology, Faculty of Psychology, Beijing Normal University, Beijing 100875, China; 2 School of Psychological Science, University of Bristol, Bristol BS8 1QU, UK; c.kent@bristol.ac.uk (C.K.); j.briscoe@bristol.ac.uk (J.B.)

**Keywords:** retrieval-induced forgetting, retrieval-induced facilitation, retrieval practice, information integration, aging

## Abstract

Retrieval practice enhances memory for practiced information, but at the price of impairing memory for unpracticed information, a phenomenon known as retrieval-induced forgetting (RIF). Evidence has shown that, for young adults, RIF can be eliminated after a long interval and when textual information is used as a memorandum. The current study aims to determine whether RIF is more durable and difficult to overcome for older adults due to their cognitive deficits. Both young and older participants completed a learning session on Day 1, during which they studied word pairs (Experiment 1) or scientific prose (Experiment 2). Then, they engaged in selective retrieval practice on Days 3, 5, or 7. Finally, they undertook a final test on Day 8. Experiment 1 showed no RIF for young but a robust RIF for older participants. Experiment 2 observed retrieval-induced facilitation for young but RIF for older participants. Although both young and older participants were encouraged to use an integration technique to facilitate learning during Experiment 2, the levels of integration only predicted the magnitudes of retrieval-induced facilitation for young but not for older participants. This study shows that older adults should be careful of carrying out selective retrieval because this may produce a more durable impairment in their memory for unpracticed information.

## 1. Introduction

Retrieval is not a neutral process. Instead, it can affect memory for both retrieved and unretrieved information. It has been documented that retrieval practice is a double-edged sword (for a review, see Roediger & Abel, 2022). On the positive side, retrieval practice can more effectively consolidate long-term memory of retrieved information, in comparison with other strategies such as note-taking, restudying, and brain mapping. This phenomenon is known as the testing effect (for reviews, see Rowland, 2014; Yang et al., 2021). However, on the negative side, retrieval practice can concurrently lead to forgetting of unretrieved information, a phenomenon known as retrieval-induced forgetting (RIF; for reviews, see Anderson, 2003; Murayama et al., 2014).

A widely utilized procedure for exploring RIF was developed by Anderson et al. (1994). During the initial learning session, participants are presented with a set of category exemplars to study (e.g., ANIMAL: monkey and tiger; FRUIT: orange and apple). Next, they engage in a practice session, during which they are asked to selectively retrieve half of the exemplars from half of the categories (e.g., ANIMAL—m____). Consequently, this research paradigm divides study items into three sub-sets, including retrieved items from practiced categories (Rp+; e.g., ANIMAL—monkey), unretrieved items from practiced categories (Rp−; e.g., ANIMAL—tiger), and unretrieved items from unpracticed categories (serving as the baseline controls, BL, e.g., FRUIT—orange; FRUIT—apple). After the practice session, participants complete a final test, during which they need to recall as many studied items as they can. The results typically show poorer recall of Rp− items than that of BL ones, even though neither Rp− nor BL items have been practically retrieved during the practice session (Anderson et al., 1994; Anderson, 2003).

A popular theory of RIF is the *inhibition account* (for a review, see Anderson, 2003). This account argues that, during the practice session, retrieval of Rp+ items would result in suppression of Rp− items in order to improve successful retrieval of the target Rp− items. Memory for Rp− items is suppressed during the practice session because these items share strong semantic relations with Rp+ items and may therefore interfere with the target retrieval process. Accordingly, this theory predicts that the magnitude of RIF should be smaller for older than for young adults (following Aslan and Bäuml (2012), we categorize younger adults as those aged 18–25 and older adults as those aged over 60) because older adults generally have deficits in inhibition, which is a core feature of cognitive aging (Hasher & Zacks, 1988). To our knowledge, only a single study by Aslan and Bäuml (2012) has tested this prediction. The study showed RIF for ‘old’ adults (i.e., 60 to 75 years old) but not for ‘older old’ ones (over 75 years old), supporting the inhibition account (Hasher & Zacks, 1988). That is, the older old adults suffered more from cognitive aging, so less inhibition of the related information was triggered when retrieving the targets.

As RIF has been confirmed to be robust for older adults aged between 60 and 75, it is meaningful to further explore how selective retrieval affects their memory in daily life. It should be highlighted that two issues of RIF have not been explored so far: (1) is RIF long-lasting for older adults? and (2) can RIF for adults be reduced or eliminated? The duration of RIF is of great practical importance. For instance, if RIF is just a temporary phenomenon and automatically disappears after a certain delay, we should not be bothered by this subtle effect on daily memory. However, if selective retrieval causes long-lasting or even permanent memory impairment, we should be very careful to use selective retrieval in daily life. Previous studies have indicated that long-lasting retrieval-induced forgetting (RIF) can pose significant challenges for older adults. For instance, it can lead to difficulties in remembering crucial health-related information, such as medication dosages or medical appointment dates. In terms of quality of life, older adults may also experience difficulties in learning new information or skills, which can result in reduced autonomy and a general decline in life quality (Passarello et al., 2022). Considering its practical importance, the present study is motivated to explore whether RIF is more durable or even permanent for older adults. If the answer is affirmative, the present study also aims to explore if information integration can be employed to reduce or even eliminate RIF for older adults.

### 1.1. Duration of RIF

It has been widely assumed that RIF is just a transient effect and memory for suppressed items normally recovers after a certain delay (e.g., after 24 h). This recovering prediction has been confirmed by many studies (e.g., Carroll et al., 2007; Chan, 2009; Saunders & MacLeod, 2002; MacLeod & Hulbert, 2011). However, many other studies found that RIF is long-lasting. For instance, it has been shown that RIF can last even more than 7 days (e.g., Garcia-Bajos et al., 2009; Migueles & García-Bajos, 2007; Storm et al., 2012). Furthermore, a meta-analysis by Murayama et al. (2014) showed that RIF, especially RIF of eyewitness memory, can be more durable.

A reason for the inconsistent findings discussed above is that the durability of RIF can be moderated by a range of factors, such as material type (Chan et al., 2006) and experiment design (Storm et al., 2012). However, none of the previous studies have investigated whether aging (young vs. older adults) moderates the durability of RIF. This question bears important practical implications for older adults, especially for those with cognitive decline. In a recent review, Marsh and Anderson (2022) proposed that the inhibition effect caused by selective retrieval may disrupt the memory consolidation process. Although this hypothesis has not yet been empirically tested, it suggests the possibility that RIF may yield a more durable impairing effect for older than for young adults because older adults are more suspectable to deterioration of sleep-dependent memory consolidation (Harand et al., 2012; Pace-Schott & Spencer, 2015; for a review, see Gui et al., 2017). A meta-analysis by Gui et al. (2017) reported that sleep is a key stage of memory consolidation for young but not for older adults, as older adults normally sleep less and their sleep quality is worse. Additionally, brain structure alteration, as a function of aging, can also induce dysfunction in memory transfer from the hippocampus to neocortex and disrupt the recovery of suppressed memory for Rp− items. Accordingly, it is reasonable to predict that RIF is more durable for older than for young adults.

### 1.2. Integration to Reverse RIF

Promisingly, previous studies showed that RIF can be reduced or even overturned by some manipulations or interventions. For example, although RIF has been reliably observed in studies employing category exemplars as study stimuli, this phenomenon is often absent when the study stimuli are changed to complex materials, such as text passages. Some studies even observed retrieval-induced facilitation by showing that selective retrieval of Rp+ textual information boosts recall of Rp− information (Chan et al., 2006; Rowland & DeLosh, 2014). A possible explanation for retrieval-induced facilitation is that processing of complex materials (e.g., text passages) normally involves a higher level of information integration, which is a resistant process to RIF. Integration refers to the process wherein related memories become interconnected in the brain through the recruitment of overlapping neuronal populations (Schlichting & Preston, 2015). Specifically, when study materials are more coherent, learners may integrate different segments of information into a unified whole (Chan, 2009; for a review, see Storm et al., 2015). In a study by Chan (2009), participants were asked to learn two articles, with sentences presented individually in their natural, coherent order, rather than learning word pairs as is common in typical RIF studies. The results indicated that retrieving information could enhance, rather than impair, the memory of related information. Interestingly, when the order of the sentences was randomized, the effect of RIFA disappeared. As the authors suggest, the use of highly cohesive materials resulted in a greater level of integration, allowing the retrieval of related positive (Rp+) items to concurrently activate memory for related negative (Rp−) items, thereby facilitating (rather than impairing) memory for Rp− items.

Previous studies have shown that individuals’ semantic integration ability deteriorates as a function of aging across adulthood (e.g., Zhu et al., 2019). That is, in comparison with young adults, older adults have deficits in integrating related information because older information integration is a resource-consuming process and older adults’ working memory capacity is typically more limited in comparison with young adults’ (Zhu et al., 2019). Also, the speed at which the brain processes information tends to slow down with age. This slowdown could make it more difficult for the elderly to efficiently integrate information during learning, making them more vulnerable to RIF. Additionally, neurological studies observed that older adults’ dysfunction in semantic integration is caused by structural changes in the brain (e.g., Zhu et al., 2017). Accordingly, it is reasonable to predict that, when taking complex materials as study stimuli, selective retrieval may generate stronger RIF for older than for young adults. Furthermore, because young adults can take further advantage of semantic integration, selective retrieval may produce retrieval-induced facilitation for Rp− items. By contrast, older adults may still suffer from RIF due to their impaired semantic integration ability. To our knowledge, no studies have explored whether selective retrieval produces stronger RIF for older than for young adults when studying complex materials. Hence, another aim of the present study is to examine this critical question.

### 1.3. The Present Study

Overall, previous studies have indicated that aging significantly mitigates the magnitude of RIF. Specifically, cognitive aging impairs the function of inhibition, leading to a diminished appearance of RIF in older adults as they age (Aslan & Bäuml, 2012). However, in most studies where RIF is used as an index of inhibitory control (e.g., Conway & Fthenaki, 2003; Storm & White, 2010), the standard paradigm involves a brief retention interval of approximately 5 min between retrieval practice and the final test. This brief interval does not allow us to observe the real RIF that might occur in real-life situations, where there might be a longer gap between retrieval practice and encoding. Given that a number of studies have found that RIF can be long-lasting (e.g., Garcia-Bajos et al., 2009; Migueles & García-Bajos, 2007; Storm et al., 2012), it is important to explore how selective retrieval affects older adults’ memory in real-life scenarios. Therefore, the present study developed a new procedure to mimic a real-life learning setting. Specifically, participants engaged in multiple retrieval practices over one week and then completed a final test one week after the initial study phase.

Considering that older adults suffer from deficits in memory consolidation and semantic integration, we predicted that, compared with young adults, older adults would show more severe memory impairment caused by selective retrieval (a larger RIF). The present study investigated this possibility from two facets. First, Experiment 1 explored whether RIF is more durable (i.e., long-lasting) for older than for young adults. Second, Experiment 2 examined whether older adults would also suffer from RIF even when studying complex text materials.

## 2. Experiment 1

The main purpose of Experiment 1 was to explore whether RIF is more durable for older than for young adults. We hypothesized that, for young adults, offline memory consolidation can eliminate the inhibition effect of selective retrieval on memory for Rp− items. However, RIF would persist after a long retention interval for older adults because aging generally causes deterioration of sleep-dependent memory consolidation.

### 2.1. Methods

#### 2.1.1. Design

A 2 × 3 mixed design was employed, with age (young vs. old) as a between-subjects factor and item type (Rp+, Rp− and BL) as a within-subjects factor.

#### 2.1.2. Participants

As the present study adopted a new procedure that is different from the standard RIF paradigm (i.e., longer interval between retrieval and test), this may introduce new variables that could influence the effect size, making estimates based on studies with standard procedures less reliable. Thus, we did not estimate the required sample size based on any previously reported effect sizes of RIF. Instead, a default medium-sized effect (Cohen’s d = 0.5) was employed.

To achieve a minimum statistical power of 0.80, the required sample size for detecting a significant (two tailed α=0.5) RIF was 34 participants per group. Due to over recruitment, the final sample size was 50 participants per group. Five older and six young adults did not complete the entire experiment: ten participants only attended the first learning session and then quit the experiment; and one participant in the older adult group did not complete the learning session. Thus, the final data analysis included 45 older (60–74 years, *M* = 69.8 years, *SD* = 2.3; 25 female) and 44 young participants (18–25 years, *M* = 21.1 years, *SD* = 1.6; 21 female). Participants received RMB 60 as task compensation. All participants signed a consent form to participate, and the protocol was ethically approved by the Faculty of Psychology, Beijing Normal University.

#### 2.1.3. Materials

*Psychopy* v2023.1.2 (Peirce, 2007) was used to implement the experiment. The stimuli were 48 Chinese category exemplars taken from eight categories (i.e., fruit, Four-footed animal, vegetables, tools, furniture, Occupations, electronic devices, and Organs). All exemplars were taken from a standardized database developed by Cai and Brysbaert (2010), which ensures their validity and reliability. The words were all medium-frequency, concrete, and imaginable nouns. All exemplars consisted of two or three Chinese characters.

For each Rp+ trial, participants were given Chinese pinyin, a standard romanization system for standard Mandarin Chinese, as prompts. Specifically, the capital letter of each syllable (Chinese character) was provided. For example, *x-n* was provided for rhino, which is a two-character Chinese word with a pronunciation of *xiniu* (i.e., 犀牛).

### 2.2. Procedure

**Figure 1:**
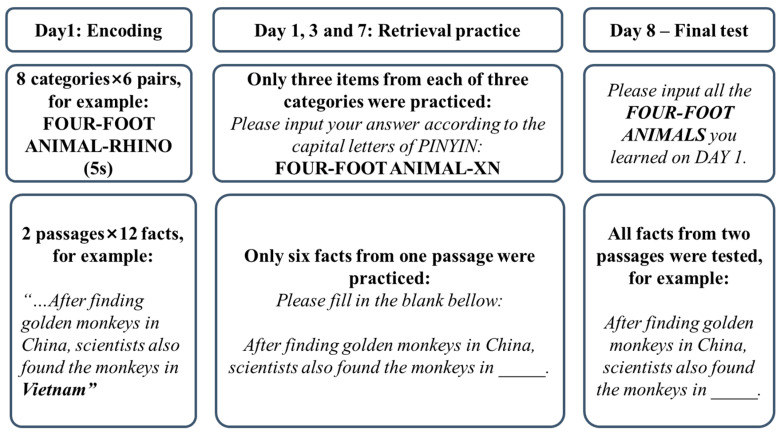
Procedures of Experiments 1 and 2.

As shown in Figure 1, the experiment lasted for 8 days, consisting of a learning session on Day 1, three practice sessions on Days 3, 5, and 7, and one final test on Day 8. The reason we set three retrieval sessions was to ensure the effects of retrieval practice were robust and enduring (Anderson, 2003). Implementing multiple practice sessions allowed us to closely mimic real-life learning environments where information retention and recall are reinforced over time. Participants in both young and old groups completed the same tasks. The learning session on Day 1 occurred in a lab in which participants learned 48 exemplars belonging to 8 categories, with 6 exemplars from each category. Following Aslan and Bäuml (2012), each category–exemplar pair was presented for 5 s at the center of the screen with a 1 s inter-stimulus interval. The presentation order of the exemplars was randomized except for the constraint that two exemplars from the same category were never presented successively.

The three retrieval practice sessions were completed online on Days 3, 5, and 7. During each retrieval practice session, participants took a cued recall test on their personal computers. In each practice test, only four categories were randomly selected, and three exemplars from each selected category were selected to form 12 Rp+ items. The category–*pinyin* pairs (e.g., *FOUR-FOOT ANIMAL-X__N__* or *FOUR-FOOT ANIMAL-RIHNO*) of the 12 Rp+ items were shown one by one in a random order at the center of the screen. Participants had 12 s to respond to each practice trial. Note that, for each participant, the Rp+ items were the same in the three practice tests.

On Day 8, participants returned to the lab and completed a final test on all items. In the final test, participants were given the category name on the top of the screen and needed to input as many studied items belonging to this category as they could. There was no time pressure and no feedback in the final test.

### 2.3. Results and Discussion

**Figure 2:**
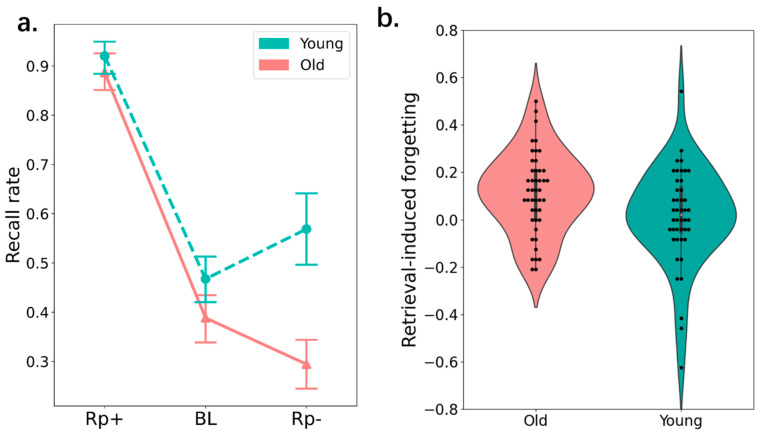
(**a**) Recall rate of older adults and young adults. (**b**) The violin plot indicates the distribution of the effect size of retrieval-induced forgetting (i.e., baseline–Rp−). Note: Error bars indicate ±1 standard error of the mean.

A 2 × 3 mixed analysis of variance (ANOVA) was performed, with group (young vs. old) as the between-subjects factor, item type (Rp+ vs. BL vs. Rp−) as the within-subjects factor, and final test performance as the dependent variable (see Figure 2). There was a main effect of age group, *F*(1, 87) = 41.92, *p* < 0.001, $\eta_{p}^{2}$ = 0.33, *BF*_10_ > 1000, with superior recall for young (*M* = 0.562, *SD* = 0.110) than for older participants (*M* = 0.43, *SD* = 0.10). There was also a main effect of item type, *F*(2, 176) = 166.04, *p* < 0.001, $\eta_{p}^{2}$ = 0.66, *BF*_10_ > 1000, with the highest recall performance for Rp+ items followed by BL items and then Rp− items.

Critically, the interaction between age group and item type was significant, *F*(2, 176) = 3.70, *p* = 0.03, $\eta_{p}^{2}$ = 0.06, *BF*_10_ = 7.94. As shown in Figure 2a,b, in both the young and older groups, recall of Rp+ items (young: *M* = 0.81, *SD* = 0.15; old: *M* = 0.72, *SD* = 0.19) was better than that of BL items (young: *M* = 0.48, *SD* = 0.17; old: *M* = 0.38, *SD* = 0.15): difference in the young group = 0.33 [0.23, 0.43], *t*(44) = 9.93, *p* < 0.001, *d* = 2.04, *BF*_10_ > 1000; difference in the older group = 0.34 [0.25, 0.44], *t*(43) = 10.53, *p* < 0.001, *d* = 2.14, *BF*_10_ > 1000. These results reflect that additional processing during the practice session substantially improved recall of Rp+ items. Importantly, in the older group, recall of Rp− items (*M* = 0.26, *SD* = 0.14) was poorer than that of BL items (*M* = 0.38, *SD* = 0.15) (young; old: *M* = 0.26, *SD* = 0.14), difference = −0.11 [−0.21, −0.02], *t*(43) = −9.93, *p* = 0.003, *d* = −0.71, *BF*_10_ = 376.60, reflecting that RIF persisted after a long delay for older adults. By contrast, in the young group, there was minimal difference in final recall performance between Rp− (*M* = 0.47, *SD* = 0.16) and (*M* = 0.48, *SD* = 0.17) BL items, difference = −0.01 [−0.11, 0.09], *t*(43) = −0.40, *p* = 0.69, *d* = −0.08, *BF*_10_ = 0.18, reflecting no RIF after a long delay for young adults.

Overall, Experiment 1 demonstrated that selective retrieval is more harmful to older adults’ memory for Rp− items. More interestingly, although for the young adults, RIF disappeared after a one week, indicating that selective retrieval is just an adaptive and temporary inhibitory control process (e.g., Carroll et al., 2007; Chan, 2009; Saunders & MacLeod, 2002; MacLeod & Hulbert, 2011), this inhibition effect survived after a relatively long period for the older adults. This might be due to older adults’ deterioration of sleep-dependent memory consolidation.

## 3. Experiment 2

Previous studies have demonstrated that coherent materials can prevent the occurrence of RIF (Chan et al., 2006; Chan, 2009). Furthermore, it has also been shown that selective retrieval can induce retrieval-induced facilitation for coherent materials due to the fact that processing of such materials requires a higher level of information integration (Chan et al., 2006). However, older adults may less be able to benefit from information integration due to their deteriorated semantic processing ability. Thus, we predicted that using more coherent materials would not eliminate the RIF of older adults.

### 3.1. Methods

#### 3.1.1. Participants

Following Experiment 1, to achieve a power of 0.80 with an effect size of *d* = 0.50, the minimum required sample size was 34 participants per group. Due to over-recruitment, we finally recruited 50 participants per group in the present experiment. Three young and five older participants who did not complete the entire experiment were excluded (i.e., only completing the learning session but not the retrieval and final test sessions), leaving final data from 45 young and 47 older participants. They were reimbursed RMB 60 for participation and signed an agreement to participate. The protocol was ethically approved by the Faculty of Psychology, Beijing Normal University.

#### 3.1.2. Materials

The learning materials were two scientific passages, one about *the Chogori* and the other about *golden monkeys*. The scientific extracts were chosen because they represent a type of complex and coherent material, which in previous studies has been shown to reduce or reverse RIF (Chan, 2009). The length of the two passages was identical, consisting of 360 Chinese characters. Each passage contained 14 sentences in total, including a starting sentence, 12 middle sentences, and an ending sentence. Each of the 12 middle sentences described a distinct fact, and there were in total 12 facts described in each passage.

### 3.2. Procedure

As is shown in Figure 1, the schedule of Experiment 2 was similar to that of Experiment 1, but with different study materials. During the learning session, participants had 15 min to read each passage. After the initial reading of Text 1, there was a three-minute break before Text 2 was displayed. The presentation order of the two passages was counterbalanced across participants. A post hoc check confirmed that the presentation order of the two passages did not affect final recall performance (*ps* > 0.05). Next, all participants completed three online practice sessions on Days 1, 3, and 7. Six knowledge points were randomly selected from one passage to act as Rp+ items. For each retrieval practice trial, participants answered a fill-in-the-blank question (e.g., *the Qogir Southwest Peak is in _____.*). There was no time pressure for retrieval practice trials and the presentation order of practice questions was randomized during each practice session. Participants received corrective feedback (i.e., correct answer) after answering each practice question. On Day 8, participants returned to the lab and finished the final test. The form of the final test was identical to the retrieval practice, but participants were tested on all knowledge points described in the two passages. The presentation order of final test questions was randomized. There was no time pressure and no feedback in the final test. After the final test, participants were asked to report the frequency of utilizing the information integration strategy during the learning and practice sessions (“*I managed to build relationships between segments of the learning materials*”: 1 = not at all; 7 = always).

### 3.3. Results and Discussion

**Figure 3:**
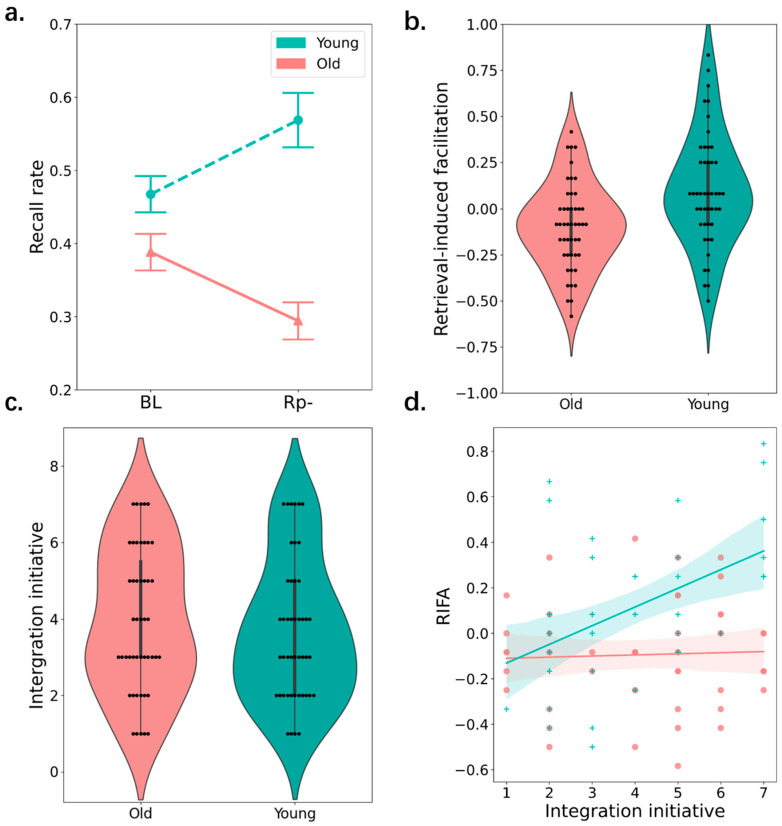
(**a**) Recall rate for baseline items and Rp− items of two age groups. (**b**) The violin plot indicates the distribution of the effect size of retrieval-induced facilitation (i.e., Rp−–baseline) of two age groups. (**c**) The violin plot indicates the distribution of the integration initiative of two age groups. (**d**) The correlation between the effect size of retrieval-induced forgetting and integration initiative for two age groups. Note: Error bars indicate ±1 standard error of the mean.

Because participants received corrective feedback after answering practice question during the practice sessions, recall of Rp+ items reached the ceiling effect in the final test for both age groups (see Figure 3a). Thus, we did not include Rp+ items in the below analyses. Instead, the below analyses focus on the key comparison between final recall of BL and Rp− items. A 2 × 2 ANOVA revealed a main effect of group, *F*(1, 91) = 41.92, *p* < 0.001, $\eta_{p}^{2}$ = 0.29 *BF*_10_ > 1000, with superior recall in the young (*M* = 0.50, *SD* = 0.14) compared to the older group (*M* = 0.36, *SD* = 0.13). But there was no main effect of item type, *F*(1, 91) = 0.02, *p* < 0.89, $\eta_{p}^{2}$ < 0.001, *BF*_10_ = 0.19.

As shown in Figure 3a,b, there was a reliable interaction between group and item type, *F*(1, 91) = 12.16, *p* < 0.001, $\eta_{p}^{2}$ = 0.12, *BF*_10_ = 49.11. Specifically, in the young group, recall of Rp− items (*M* = 0.57, *SD* = 0.25) was better than that of BL items (*M* = 0.47, *SD* = 0.17), difference = 0.10 [−0.01, 0.21], *t*(44) = 2.55, *p* = 0.038, *d* = 0.52, *BF*_10_ = 1.61, reflecting a retrieval-induced facilitation effect for young participants. By contrast, in the older group, recall of Rp− items (*M* = 0.29, *SD* = 0.17) was better than that of BL items (*M* = 0.39, *SD* = 0.17), difference = −0.09 [−0.20, 0.01], *t*(45) = 2.38, *p* = 0.038, *d* = 0.48, *BF*_10_ = 4.59, reflecting a RIF effect for older adults.

Next, we evaluated the role of semantic integration in the effect of selective retrieval on memory for Rp− items (the magnitude of the effect of selective retrieval on memory for Rp− items was calculated as the difference in final recall between Rp− and BL items, with positive values representing retrieval-induced facilitation and negative values representing RIF) (i.e., the effect size of RIFA). There was minimal difference in integration strategy usage between the young (*M* = 3.67, *SD* = 1.85) and older (*M* = 3.94, *SD* = 1.87) groups, difference = 0.27, *t*(44) = 1.09, *p* = 0.44, *d* = 0.17, *BF*_10_ = 0.66, reflecting that young and older participants did not differ in integration strategy usage (see Figure 3c). A linear regression was established, with age group and semantic integration usage as independent variables and the effect size of RIFA as dependent variable. As shown in Figure 3d, there was a significant interaction between group and integration strategy usage on retrieval-induced facilitation, *b* = 0.06 [0.04, 0.08], *p* = 0.009. That is, integration strategy usage positively predicted the effect of selective retrieval on memory for Rp− items for young participants, *b* = 0.10 [0.01, 0.19], *p* < 0.001, but this pattern was not observed for older participants, *b* = 0.08 [0.02, 0.14], *p* = 0.01. These results suggest that the more frequently young participants utilized the integration strategy, the more effectively selective retrieval facilitated recall of Rp− items. However, this facilitation effect on memory for Rp− items induced by semantic integration was absent for older participants.

Overall, Experiment 2 confirmed another negative effect of selective retrieval on older adults’ memory for Rp− items: Older adults still suffer from RIF even when they study more cohesive study materials. By contrast, young adults’ memory for Rp− items benefits from selective retrieval when studying cohesive materials. More interestingly, younger and older participants reported a well-matched level of integration strategy usage, but equivalent usage of semantic integration produced differential mnemonic outcomes. This difference may result from older adults’ deficits in semantic integration (Zhu et al., 2017, 2019), even when they utilized this strategy as frequently as young adults. Put differently, it is possible that even though older adults actively try to integrate different pieces of information, their memory cannot really benefit from this integration process due to cognitive decline in this ability.

## 4. General Discussion

Across two experiments, the present study highlighted more pronounced detrimental effects of selective retrieval for older adults’ memory for unpracticed information. Specifically, though RIF was eliminated after a long retention interval for young adults, the suppressed memory for Rp− items did not recover for older adults (Experiment 1), suggesting that RIF is more durable for older than for young adults. Moreover, when more coherent materials (i.e., scientific passages) were used as study stimuli, selective retrieval led to a facilitation effect on memory for Rp− items for young adults but a RIF effect for older ones (Experiment 2). Thus, in spite of abundant evidence indicating that RIF is merely an adaptive and transient inhibition process (e.g., Carroll et al., 2007; Chan, 2009; Saunders & MacLeod, 2002; MacLeod & Hulbert, 2011) and can be reduced by integration (e.g., Chan, 2009), the present study established that RIF could persist stubbornly in older adults. Even though inhibition deficits are often viewed as a core feature of cognitive aging, this does not mean that older adults are immune to RIF. The present findings aligned with those of Aslan and Bäuml (2012) by showing that older adults, aged between 60 and 75 years old, are still susceptible to RIF.

The findings documented here contribute to the body of literature addressing deficits in long-term memory consolidation caused by aging. As is suggested by Aslan and Bäuml (2012), executive functions, such as inhibition and interference control, are essential for proper memory functioning and decline with age. Thus, aging may make it more difficult to suppress irrelevant information (Rp− items) during selective retrieval, leading to greater RIF for older adults. Marsh and Anderson (2022) theorized that inhibition induced by selective retrieval could impair memory consolidation and lead to permanent forgetting. Lending credence to this hypothesis, the present study showed that such disruption does occur, at least among the older population. Therefore, while numerous studies have suggested that RIF is generally a temporary inhibition process and the memory for Rp− items can become recallable (released from inhibition) after a long delay for young adults, our findings demonstrate that RIF was rather durable for older adults. To our knowledge, the present study is the first to demonstrate that age moderates the duration of RIF.

Aside from deficits in memory consolidation, deficits in semantic integration may also render older adults more susceptible to RIF. An interesting finding observed here was that young and older participants differed minimally in the self-reported usage of the integration strategy. However, only for young participants did the usage of the integration strategy positively correlate with the effect of selective retrieval on memory for Rp− items. This suggests the older adults’ ineffectiveness during encoding, possibly caused by alterations in brain structure with age (Zhu et al., 2017, 2019), even if older adults have managed to use the strategy of integration. Overall, the present study upholds the view that information integration plays a critical role in determining whether selective retrieval facilitates or impairs memory for Rp− information.

The present study provides an important implication that selective retrieval practice may result in persistent memory impairments for specific populations or under specific circumstances. For instance, research has demonstrated that both short-term deprivation and long-term sleep disorders can result in memory consolidation dysfunction (Stickgold, 2005). Consequently, selective retrieval may produce permanent memory loss in these instances. This information carries critical implications for real-world educational practices, such as the common occurrence of sleep deprivation and selective retrieval when students are preparing for high-stakes course exams. In this case, it is crucial to understand that selective retrieval may lead to the permanent forgetting of unpracticed information, especially when they suffer from poor sleep quality (and poor memory consolidation) during the exam preparation phase. Similarly, individuals with impaired semantic integration, such as some autistic individuals (e.g., McCleery et al., 2010), dyslexia (Schulz et al., 2008), and ADHD (e.g., Tannock et al., 2000), may also experience RIF after a prolonged retention interval. However, notably, these individuals may also exhibit inhibitory control deficits that could yield the potential to reduce RIF. We encourage future research to empirically examine how selective retrieval affects memory for unpracticed information among the aforementioned populations.

Although the present study demonstrated that older adults are more susceptible to RIF compared with young adults, existing evidence has proposed approaches to avoid or mitigate RIF. For example, the long-term RIF effect can be eliminated by providing individuals with an intermittent relearning opportunity, namely re-exposure to Rp− items (Storm et al., 2012). Additionally, a core component of RIF is retrieval dependence (Bäuml & Aslan, 2004). That is, if memory for Rp+ items is enhanced by restudying, no RIF would occur, as restudying Rp+ items does not necessarily need to resolve the interference from competitors (i.e., Rp− items). Although replacing retrieving with restudying abolishes the benefits of test-enhanced learning (Rowland, 2014; Yang et al., 2021), it avoids the detrimental effect on unpracticed information. Furthermore, by realizing that selective retrieval produces a more severe and more permanent impairment effect on memory for unpracticed information for older adults, the optimal strategy for older adults is to recall all contents (rather than selectively recall part of them), as test-enhanced learning has been shown to be robust in older adults (Rowland, 2014).

### 4.1. Limitations and Future Directions

The first limitation of the present study is that we did not include an immediate test as in most previous RIF studies, so we did not measure any interaction between RIF (or retrieval-induced facilitation) and time. The lack of an immediate test limits the ability to assess the evolution of RIF over time and to compare the results with previous studies that used an immediate test (i.e., the standard RIF paradigm). For example, Chan (2009) found that retrieval-induced facilitation emerged after 24 h but not after 20 min. Thus, there might be a possibility that the young and old groups would produce a similar pattern if the final test was administered immediately after the (selective) retrieval practice session. A possible direction for future research could be a longitudinal study, which would offer a more comprehensive observation of the interaction between RIF and time. Secondly, the present study only recruited younger-old adults ranging from 60 to 70 years old. However, as demonstrated by Aslan and Bäuml (2012), RIF was eliminated for the older-old adults over 75 years old. Nonetheless, Aslan and Bäuml (2012) only explored RIF on memory for word pairs; future research can profitably explore whether selective retrieval produces different effects for these two age groups with complex materials (e.g., text passages) as study stimuli. It is possible that the older-old adults may resist RIF due to their inhibition deficits, and also resist retrieval-induced facilitation due to their integration deficits. Finally, we propose that deficits in memory consolidation and semantic integration may be the cause of more durable RIF and the disappearance of retrieval-induced facilitation for older adults. However, these proposals are not rigorously tested here. Thus, future studies are encouraged to investigate these dynamics more directly. For example, employing neurological approaches could help determine whether the structures related to semantic integration are less involved during encoding and retrieval in older adults. This approach could provide deeper insights into how aging affects inhibitory control and memory integration over extended periods.

### 4.2. Concluding Remarks

RIF is a critical concern for older adults due to their deficits in memory consolidation and semantic integration. Different from younger adults, selective retrieval causes long-term (or potentially permanent) memory impairment for older adults. Additionally, information integration protects young adults from RIF but fails to help the older adults resist RIF, even if both young and older adults manage to use this strategy during the study phase. Overall, we raise the caveat that testing, which is considered to be an effective strategy for young adults, may not be the optimal choice for older adults when no sufficient time is available for retrieving all studied information during the practice phase. This risk of selective retrieval should be considered when designing educational and healthcare plans for older adults.

## Data Availability

All the data are available from the OSF link: https://osf.io/3czqr/?view_only=e7d6ea6f9e174b06b794d660e4b1814e (accessed on 5 February 2025).
